# Influence of ABC stroke score on late recurrence of paroxysmal atrial fibrillation following radiofrequency catheter ablation

**DOI:** 10.1186/s13019-024-02847-z

**Published:** 2024-06-21

**Authors:** Ke-Zeng Gong, Zhe Xu, Ting-Pei Zhuang, Xue-Hai Chen, Jian-Hua Chen, Wei-Wei Wang, Wen-Hua Xu, Fei-Long Zhang

**Affiliations:** 1 Department of Cardiology, Fujian Medical University Union Hospital; Fujian Heart Medical Center; Fujian Institute of Coronary Heart Disease; Fujian Clinical Medical Research Center for Heart and Macrovascular Diseases, No.29 Xin-Quan Road, Gulou District, Fuzhou, 350001 Fujian China; 2 Department of Cardiology, The First Hospital of Quanzhou, Quanzhou, 362000 Fujian China; 3 Department of Cardiology, Changji Prefecture People’s Hospital in Xinjiang Uygur Autonomous Region, No.303 Yan-an Road, Changji City, 831100 Xinjiang China

**Keywords:** ABC stroke score, Ablation, Atrial fibrillation, Late recurrence, Propensity score matching

## Abstract

**Background:**

In this study we investigated the impact of ABC stroke score on the recurrence of paroxysmal atrial fibrillation (PAF) following radiofrequency catheter ablation (RFCA).

**Methods:**

A total of 132 patients with PAF who underwent RFCA from October 2018 to September 2019 were included in this study. During the first phase of this study the patients were categorized into two groups based on late recurrence of atrial fibrillation after RFCA. In the second phase, the patients were further divided into two groups based on whether their ABC stroke score was ≥ 6.5.

**Result:**

The univariate analysis indicated that the risk factors for late recurrence of PAF included early recurrence, ABC stroke score, CHA2DS2-VASc score, and NT-proBNP (*P* < 0.05). Cox multivariate regression analysis revealed that ABC stroke score (*P* = 0.006) and early recurrence (*P* = 0.000) were independent predictors of late recurrence, and ABC stroke score ≥ 6.5 was a risk for predicting recurrence of PAF after RFCA with a sensitivity of 66.7% and specificity of 65.7%. After the completion of the 1:1 matching, the univariate Cox analysis indicated that an elevated score of ABC stroke (≥ 6.5) was an independent predictor of late recurrence of PAF (HR = 2.687, 95% CI: 1.036–6.971, *P* = 0.042). However, using an ABC stroke score cut off at 6.4 predicted the recurrence of atrial tachyarrhythmia with 85% sensitivity and 58.5% specificity.

**Conclusion:**

An ABC stroke score ≥ 6.4 is a predictor for late recurrence of PAF after RFCA.

## Background

Atrial fibrillation (AF) is one of the most prevalent clinical arrhythmia, a trend that continues to rise due to the aging population and the increasing prevalence of chronic conditions such as hypertension and diabetes. Catheter ablation has evolved into the primary treatment for drug-resistant AF benefiting from ongoing enhancements in both techniques and theories [1]. Despite the growing success rates associated with catheter ablation for AF the potential for AF recurrence persists [2]. The procedure is not only financially demanding but also recurrence following ablation can diminish the overall quality of life and increase the incidence of adverse cardio-cerebrovascular events [3]. Therefore, exploring the predictors of recurrence post AF ablation serves as a crucial step in informed clinical decision-making and enhancing the overall success rate of the procedure.

In recent years, there has been a growing focus not only on independent risk factors but also on novel scoring systems designed to predict the risk of recurrence after AF ablation. Mulder et al. [4] discussed the predictive value of 10 scoring systems for AF recurrence post catheter ablation. Their findings highlighted shortcomings in these systems, such as low predictive efficacy or a lack of combination of biomarkers and clinical indicators. Therefore, the development of a new scoring system holds significant clinical importance.

**Fig. 1:**
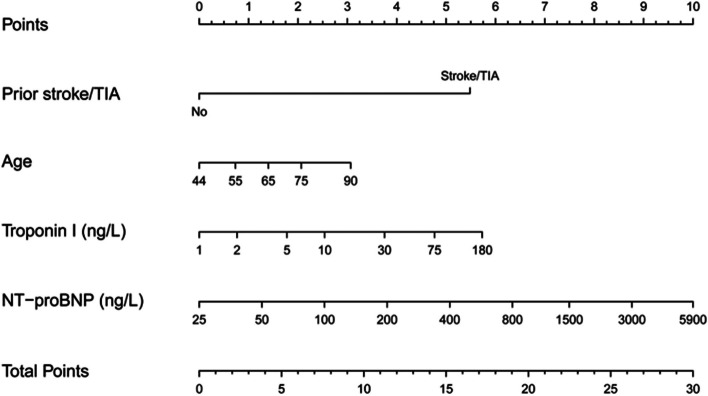
ABC stroke score form adapted from Hijazi et al. with permission

The ABC stroke score, a novel stroke risk score developed by Hijazi et al. [5], is based on age, biomarkers, and clinical history. This score was formulated using a large cohort from the ARISTOTLE trial (Fig. 1) and incorporates clinical indicators and biological markers strongly associated with AF. To date, no study has investigated the impact of ABC stroke score on the recurrence of paroxysmal atrial fibrillation (PAF) after radiofrequency catheter ablation (RFCA). Therefore, in this study we investigated the predictive value of ABC stroke score in late recurrence of PAF after RFCA.

## Methods

### Study participants

A total of 132 individuals who had undergone initial radiofrequency catheter ablation for symptomatic PAF between October 2018 and September 2019 at the Fujian Medical University Union Hospital were enrolled in this study. All patients underwent esophageal ultrasound or left atrial computed tomography angiography (CTA) examination before the catheter ablation procedure to rule out thrombosis in the left atrium and/or left atrial appendage and valvular AF. All antiarrhythmic drugs were discontinued for at least five half-lives before the procedure. Prior to undergoing treatment all the study participants provided informed consent for their participation in the study.

Patients were excluded from this study based on the following criteria: 1. age < 18 years or 80 years; 2. Patients with New York Heart Association functional class IV; 3. Patients with valulaer AF; 4. Patients with congenital heart disease, cardiomyopathy, acute coronary syndrome, pulmonary embolism, chronic obstructive pulmonary disease, severe liver or kidney insufficiency, and uncontrolled and unstable hyperthyroidism; 5. Patients left atrium and/or left atrial appendage with thrombus; 6. Patients who underwent surgery or had combined neurological diseases such as cerebrovascular accidents within the last three months; 7. Patients with incomplete clinical data.

### Radiofrequency ablation technique of AF

Experienced operators conducted all the catheter ablation procedures. Intracardiac electrograms were recorded using the Bard EP Recording System. Patients were placed under deep sedation and analgesia through continuous infusion of midazolam and fentanyl. One adjustable curved multipolar electrode was positioned in the coronary sinus via the left femoral vein following bilateral femoral venous punctures. Thereafter, Preface sheaths were introduced into the left atrium using transseptal puncture technique via the right femoral vein. Unfractionated heparin (100 U/kg) was administered directly via the Preface sheath to maintain an activated clotting time of 300 to 350 s during the procedure after transseptal puncture. The operators then performed mapping and ablation with the guidance of an electroanatomical mapping system (Carto6®; BiosenseWebster, Diamond Bar CA, USA). The endpoint of RFCA was the disconnection of the pulmonary vein from the atrium and atrial blowout stimulation failing to induce < 30 s of rapid atrial arrhythmia.

### Follow-up

Oral anticoagulants (rivaroxaban 15–20 mg qd or dabigatran 110 mg bid) were administered for a duration of least 3 months following RFCA, and an anticoagulant strategy was continued according to the patient’s risk of thrombosis assessed by CHA2DS2-VASC score. Propafenone, metoprolol or amiodarone were administered for episodes of AF within the initial 3-month postoperative period. A 72-h Holter monitoring was conducted at 3, 6, and 12 months after the procedure and an ECG was performed whenever symptoms were present. Late recurrence of AF is defined as any atrial tachycardia that lasted for more than 30 s on 24-h Holter monitoring or documented on a 12-lead ECG after a 3-month postoperative period, while early recurrence was within the first three months.

### Statistical analysis

Statistical analysis was performed utilizing the SPSS 26.0 software. Count data are expressed as frequency (percentage) and analyzed with the chi-squared test. Measurement data were tested for normality and normally distributed data are expressed as mean ± standard deviation (x ± s), with a comparison of two groups conducted using the independent samples *t*-test. Non-normally distributed data were expressed as median (25–75 quartiles) and nonparametric tests were applied. The receiver operating characteristic (ROC) curve was employed to evaluate the predictive value of the ABC stroke score in anticipating AF recurrence after RFCA. Kaplan–Meier survival analysis was performed for the primary end point and ABC stroke score. Univariate logistic regression analysis was utilized to analyze the ABC stroke score after propensity score matching. Statistical significance was determined at *P* < 0.05. Furthermore, *P* < 0 0.1 was considered statistically significant in the propensity score matching to address interference factors between the 2 groups based on whether the ABC stroke score was ≥ 6.5 before it was matched.

## Results

### Baseline according to the atrial fibrillation recurrence after catheter ablation

A preliminary RFCA was conducted for the 132 patients with symptomatic PAF from October 2018 to September 2019 and a follow-up was conducted. Among these patients, 56 (42%) were female. The average age of these patients was 62 years and the mean follow-up duration was 8 months (ranging from 6.0 to 10.8 months). Among them, 16 patients (12.1%) experienced early recurrence and 27 patients (20.5%) experienced late recurrence (Table 1).

**Table 1:** Baseline according to the atrial fibrillation recurrence after catheter ablation

	Total (*n* = 132)	Recurrence		*P* value
		Yes(*n* = 27)	No(*n* = 105)	
Clinical characteristics				
Female	56(42%)	55.6%	39%	0.122
Age(years)	62(56–68)	62(55–68)	62(59–71)	0.239
BMI^(Kg/m2)^	23.69 ± 1.85	23.61 ± 1.90	23.72 ± 1.85	0.783
Course of PAF(months)	24(6–36)	36(6–48)	24(6–36)	0.662
Hypertension	63(47.7%)	16(59.3%)	47(44.8%)	0.179
Diabetes mellitus	15(11.4%)	4(14.8%)	11(10.5%)	0.769
Hyperlipemia	56(42.4%)	8(29.6%)	47(44.8%)	0.179
Stroke/TIA	11(8.3%)	3(11.1%)	8(7.6%)	0.845
ABC stroke score	5.2(3.0–8.1)	7.7(4.0–10.1)	4.3(2.5–7.5)	0.004*
CHA2DS2-VASc	2.0(1.0–2.0)	2.0(1.0–3.0)	1.0(1.0–2.0)	0.033*
Blood Test				
NT-proBNP(pg/ml)	107(53–316)	200(75 -612)	104(48–257)	0.020*
TnI(ug/L)	0.001(0.001–0.01)	0.01(0.001–0.02)	0.001(0.001–0.01)	0.101
TG(mmol/L)	1.39(1.04–1.93)	1.32(1.04–1.71)	1.39(1.03–1.94)	0.650
TC(mmol/L)	4.24 ± 0.98	4.25 ± 1.04	4.24 ± 0.97	0.957
HDL-C(mmol/L)	1.16(0.94–1.43)	1.23(1.01–1.55)	1.15(0.93–1.36)	0.185
LDL-C(mmol/L)	2.81 ± 0.89	2.79 ± 0.83	2.82 ± 0.91	0.873
Scr(umol/L)	75.25 ± 18.08	76.93 ± 17.72	74.82 ± 18.23	0.587
Echocardiographic				
LAD(cm)	3.65 ± 0.50	3.76 ± 0.49	3.62 ± 0.51	0.203
LVEDD(cm)	4.47(4.22–4.80)	4.49(4.22–4.85)	4.46(4.22–4.75)	0.877
LVEF(%)	62(60–65)	62(60–65)	4.46(4.22–4.75)	0.989
Postoperative AF			62(60–65)	
Amiodarone therapy	28(21.21%)	6(22.2%)	22(21.0%)	0.886
Propafenone therapy	13(9.84%)	3(11.1%)	10(9.5%)	1.000
Follow up time (months)	8.0(6.0–10.8)	9.0(7.0–12.0)	8.0(6.0–10.0)	0.125
ERAA	16(12.1%)	12(44.4%)	4(3.8%)	0.000*

*BMI* Body mass index, *ERAA* Early recurrence of atrial arrhythmia, *HDL-C* High-density lipoprotein cholesterol, *HTHD* Hypertensive heart disease, *LAD* Left atrial diameter, *LDL-C* Lowdensity lipoprotein cholesterol, *LVEF* Left ventricular ejection fraction, *TC* Total cholesterol, *TG* Triglyceride, *LVEDD* left ventricular end-diastolic dimension

^*^p < 0.05. Data given as mean ± SD,n(%),or median (interquartile range)

### Univariate analysis

The patients were divided into two groups on the basis of late recurrence of AF following RFCA (Table 1). The univariate analysis revealed that early recurrence (*P* = 0.000), ABC stroke score (*P* = 0.004), CHA2DS2-VASc (*P* = 0.033), and NT-proBNP (*P* = 0.020) were risk factors that affect the late recurrence of AF (*P* < 0.05). Patients with AF recurrence had a higher score of ABC stroke (7.7(4.0–10.1) vs 4.3(2.5–7.5) *P* = 0.004) and higher score of CHA2DS2-VASc (2.0(1.0–3.0) vs1.0(1.0–2.0) *P* = 0.033).

### Multiple Cox regression analysis

**Fig. 2:**
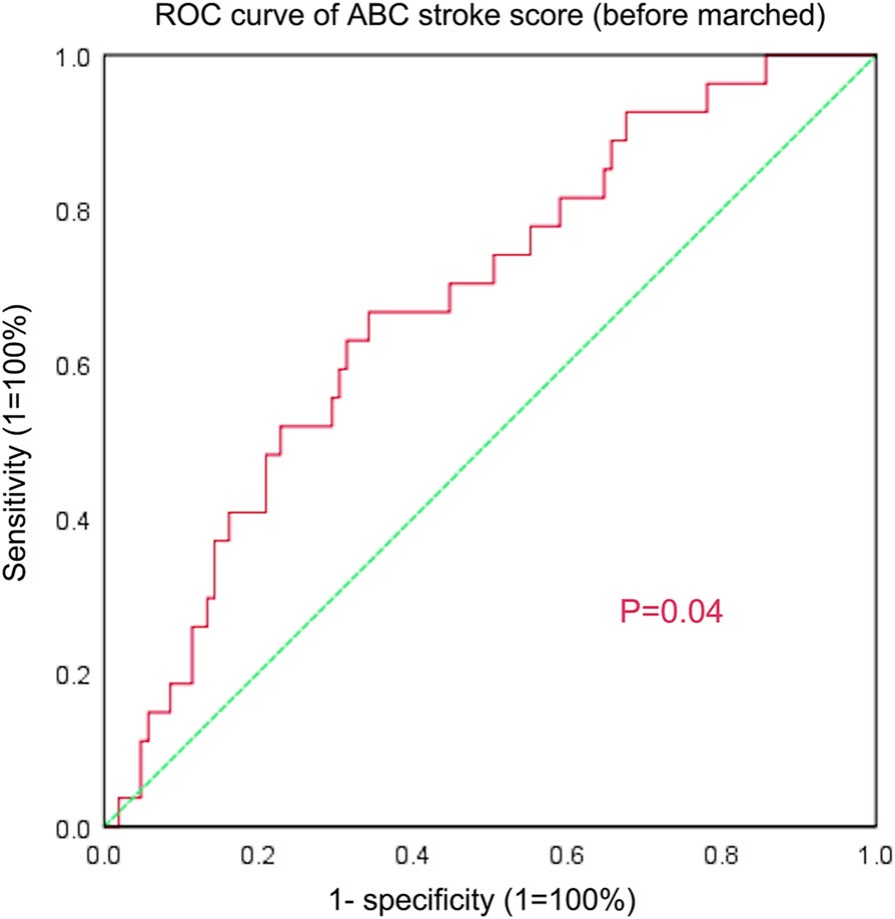
ROC curve for the ABC stroke score (Before matching)

Early recurrence, ABC stroke score, CHA2DS2-VASc score, and NT-proBNP were included in the multifactorial Cox regression analysis. The results indicated that ABC stroke score (HR: 1.121, 95% CI: 1.034–1.215, *P* = 0.006) and early recurrence (HR: 16.200, 95% CI: 6.673- 39.329, *P* = 0.000) were independent risk factors for AF recurrence (Table 2). The area under the ROC curve (AUC) for ABC stroke score (Fig. 2) was 0.678 (*P* = 0.04 95% CI: 0.570–0.786) with an optimal cut-off value of 6.5 (sensitivity 66.7%, specificity 65.7%).

**Table 2:** Multivariate Cox regression analysis for the predictors of atrial fibrillation recurrence

	Multivariate analysis	
	HR(95%CI)	*P* value
ABC stroke score	1.121(1.034–1.215)	0.006^*^
ERRA	16.200 (6.673–39.329)	0.000^*^
NT-proBNP	/	0.606
CHA2DS2-VASc	/	0.151

^*^p < 0.05. *ERAA* Early recurrence of atrial arrhythmia

### Baseline characteristics according to ABC stroke score (before matching)

All patients were divided into two groups according to the ABC stroke score: the high group (ABC stroke score ≥ 6.5) and the low group (ABC stroke score < 6.5) (Table 3). The results showed that there were significant differences in left atrial diameter and early recurrence of AF between the two groups (*P* < 0. 1).

**Table 3:** Characteristics according to whether ABC stroke score ≥ 6.5(Before marched)

	ABC ≥ 6.5 (*n* = 54)	(ABC < 6.5) (*n* = 78)	*P* value
Clinical characteristics			
Female	24(44.4%)	32(41.0%)	0.696
BMI(Kg/m2)	23.7 ± 2.1	23.7 ± 1.7	0.841
Course of PAF(months)	24.00(6.75–39.00)	12.00(6.00–39.00)	0.434
Hypertension	30(55.6%)	33(42.3%)	0.134
Diabetes mellitus	8(14.8%)	7(9.0%)	0.299
Hyperlipemia	21(38.9%)	34(43.6%)	0.590
Blood Test			
TG(mmol/L)	1.35(1.07–1.86)	1.45(1.01–1.95)	0.645
TC(mmol/L)	4.2 ± 1.1	4.3 ± 0.9	0.611
HDL-C(mmol/L)	1.17(0.95–1.52)	1.17(0.93–1.42)	0.946
LDL-C(mmol/L)	2.8 ± 0.9	2.8 ± 0.9	0.817
Scr(umol/L)	75 ± 20	75 ± 16	0.904
Echocardiographic			
LAD(cm)	3.77 ± 0.56	3.57 ± 0.45	0.021^*^
LVEDD(cm)	4.47(4.29–4.84)	4.46(4.22–4.75)	0.684
LVEF(%)	61.50(58.00–65.25)	62.50(60.00–64.85)	0.243
Postoperative AF			
Amiodarone therapy	14(25.9%)	14(17.9%)	0.270
Propafenone therapy	6(11.1%)	7(9.0%)	0.685
Follow up time (months)	8.50 (6.00–11.25)	8.00(6.00–10.00)	0.573
ERAA	10(18.5%)	6(7.7%)	0.061^*^

*BMI* Body mass index, *HDL-C* High-density lipoprotein cholesterol, *LDL-C* Lowdensity lipoprotein cholesterol, *LAD* Left atrial diameter, *LVEF* Left ventricular ejection fraction, *TC* Total cholesterol, *TG* Triglyceride, *LVEDD* Left ventricular end-diastolic dimension, *ERAA* Early recurrence of atrial arrhythmia

^*^p < 0.1. Data given as mean ± SD, n(%), or median (interquartile range)

### Baseline characteristics according to ABC stroke score (after matching)

**Fig. 3:**
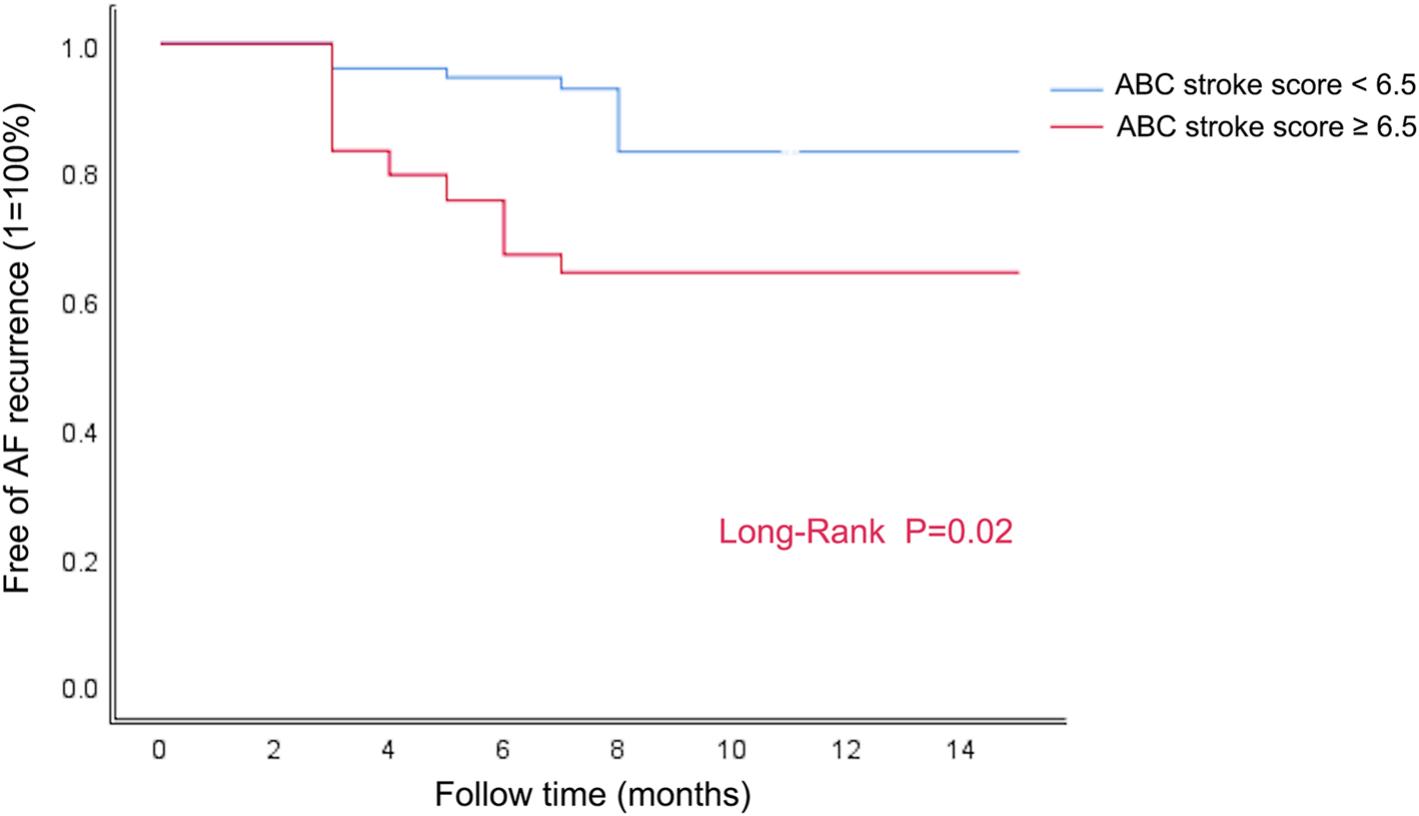
Kaplan–Meier event-free analysis for patients with the ABC stroke score ≥ 6.5 compared with patients with ABC stroke score < 6.5

**Fig. 4:**
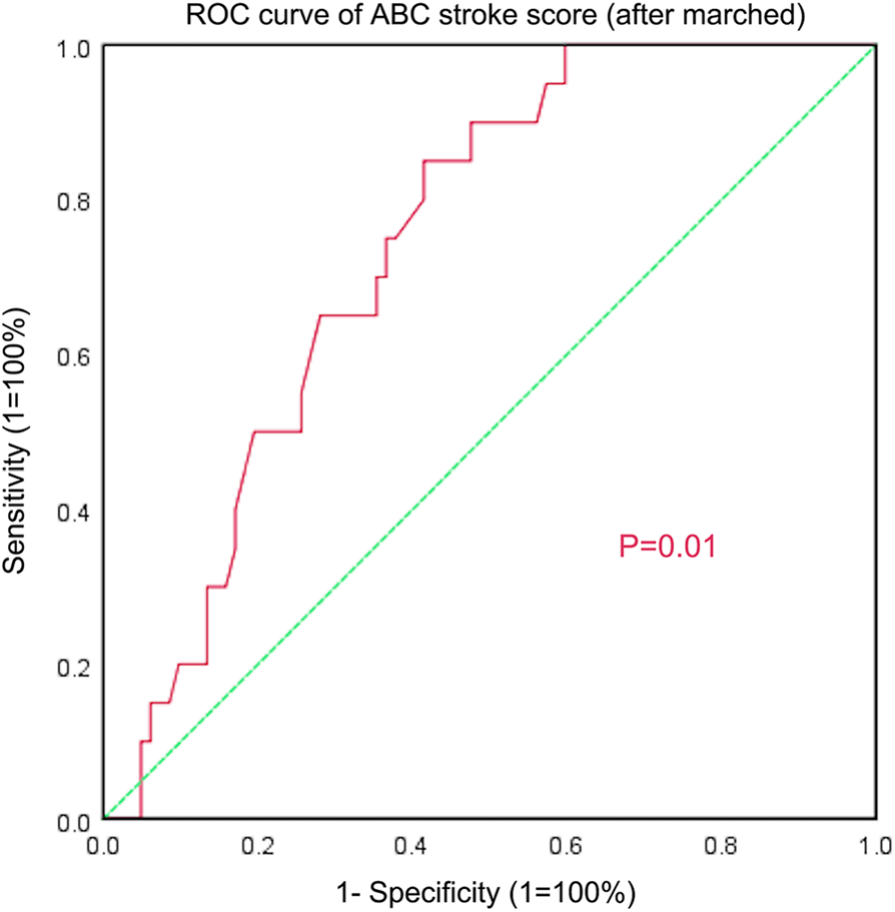
ROC curve for the ABC stroke score (After marching)

There were no significant differences between the two groups (Table 4, *P* > 0.05) following matching. Kaplan–Meier analysis showed that patients with ABC stroke score ≥ 6.5 had a higher risk of recurrence after AF ablation (*P* = 0.001) (Fig. 3). Univariate logistic regression analysis showed that ABC stroke score ≥ 6.5 was a risk factor for AF recurrence (HR = 2.687, 95% CI:1.036–6.971) (Table 5), indicating that patients with ABC stroke score ≥ 6.5 had a 2.687-fold increased chance of experiencing recurrent AF. The AUC for the matched ABC stroke score was 0.742 (95% CI: 0.643–0.842) with an optimal cut-off value of 6.4 (sensitivity 85.0%, specificity 58.5%) (Fig. 4).

**Table 4:** Characteristics according to whether ABC stroke score ≥ 6.5(After marched)

	After marched		*P* value
	ABC ≥ 6.5 (*n* = 51)	ABC < 6.5 (*n* = 51)	
Clinical characteristics			
Female	21(41.2%)	20(39.2%)	0.840
BMI(Kg/m2)	23.6 ± 2.0	23.8 ± 1.7	0.740
Course of PAF(months)	24.00(6.00–36.00)	12.00(6.00–36.00)	0.566
Hypertension	27(52.9%)	23(45.1%)	0.428
Diabetes mellitus	8(15.7%)	3(5.9%)	0.110
Hyperlipemia	19(37.3%)	22(43.1%)	0.545
Blood Test			
TG(mmol/L)	1.34(1.08–1.74)	1.58(1.07–1.95)	0.337
TC(mmol/L)	4.2 ± 1.1	4.2 ± 0.9	0.737
HDL-C(mmol/L)	1.14 (0.95–1.52)	1.13(0.90–1.43)	0.527
LDL-C(mmol/L)	2.8 ± 0.9	2.8 ± 0.9	0.905
Scr(umol/L)	75 ± 21	74 ± 17	0.740
Echocardiographic			
LAD(cm)	3.74 ± 0.54	3.63 ± 0.45	0.249
LVEDD(cm)	4.49(4.31–4.83)	4.57(4.22–4.82)	0.735
LVEF(%)	62.00(58.00–65.00)	63.00(60.00–65.00)	0.192
Postoperative AF			
Amiodarone therapy	13(25.5%)	7(13.7%)	0.135
Propafenone therapy	6(11.8%)	6(11.8%)	1.000
Follow up time (months)	9.00(6.00–12.00)	7.00(6.00–9.00)	0.055
ERAA	9(17.6%)	6(11.8%)	0.402

Data given as mean ± SD, n(%), or median (interquartile range)

*BMI* Body mass index, *HDL-C* High-density lipoprotein cholesterol, *LDL-C* Lowdensity lipoprotein cholesterol, *LAD* Left atrial diameter, *LVEF* Left ventricular ejection fraction, *TC* Total cholesterol, *TG* Triglyceride, *LVEDD* Left ventricular end-diastolic dimension, *ERAA* Early recurrence of atrial arrhythmia

**Table 5:** Univariate Logistic regression analysis for the predictors of atrial fibrillation recurrence

	*P* value	HR (95%CI)
ABC strok score ≥ 6.5	0.042	2.687(1.036–6.971)

## Discussion

To our knowledge, this is the first study to investigate the impact of ABC stroke score on the late recurrence of PAF following radiofrequency catheter ablation. The key findings of our study include the novel exploration of the predictive value of the ABC stroke score in assessing recurrence after AF ablation. Specifically, an ABC stroke score of ≥ 6.4 indicated increased risk of recurrence following RFCA of AF.

### Atrial fibrillation risk scoring system and recurrence after atrial fibrillation ablation

There is a gradual increase in the incidence of AF, posing clinical hazards that primarily include heart failure and stroke [1]. Despite advancements in catheter ablation technology and expertise there still remains a certain recurrence rate after RFCA [2]. Consequently, the latest guidelines from the European Society of Cardiology recommend evaluating not only the procedural risk but also the risk factors for AF recurrence after catheter ablation prior to the procedure. Previous studies have indicated that age, diabetes mellitus, biological indicators, and left atrial size are individual risk factors for the late recurrence after RFCA. However, these independent risk factors exhibit a compounding effect and interact with each other. Therefore, the combination of multiple related risk factors into a single quantitative risk score system may provide a more reliable assessment of the outcome following AF ablation. Presently, the commonly used risk scoring systems for AF include APPLE [6], ATLAS [7], BASE-AF2 [8], CAAP-AF [9], CHA2DS2-VASc [10], and MB-LATE [11], among others. A study conducted by Mark compared the efficacy of various scoring systems in predicting AF recurrence after catheter ablation [4]. The results indicated that these scoring systems have low predictive value for recurrence (area under curve [AUC] 0.553–0.669), although statistically significant. In our study, we observed, that the ABC stroke score had a predictive value for AF recurrence after RFCA, demonstrating a high diagnostic efficacy (AUC 0.742). The ABC stroke score is a new stroke risk score established by Hijazi et al. from a large cohort of 14,701 patients in the ARISTOTLE trial. It combines cardiac biological indicators such as NT-ProBNP and troponin with clinical indicators of age and stroke history, which are not included concurrently in other scoring systems. NT-proBNP and troponin are used as cardiac biomarkers within the ABC stroke score, representing left ventricular hypertrophy and myocardial injury associated with elevated pressure and structural remodeling of the left atrium [12].

Some studies have suggested that biomarkers such as NT-proBNP and troponin are independently associated with AF recurrence [13]. However, upon analysis, we found that NT-proBNP and troponin were not risk factors for late recurrence of AF after catheter ablation (*P* > 0.05). Additionally, contrary to other findings, age was not identified as a risk factor for late recurrence of AF after catheter ablation. This difference could be attributed to the relatively lower age of the population in this study, with participants ranging from 56 to 68 years in age, with an average age of 62 years. Furthermore, the efficacy and safety of RFCA in patients with a history of stroke have been reported to be comparable to those without such a history [14, 15]. In this study we did not find an association between a history of stroke and late recurrence of AF, also no association was found between a history of stroke and late recurrence of AF after catheter ablation. The ABC stroke score utilized in our study, which combines the above-mentioned indicators, provides a comprehensive evaluation of the clinical characteristics of patients and demonstrates predictive value in AF recurrence after catheter ablation. This is in contrast to single clinical indicators or biological markers, which exhibit weak early warning signs for late recurrence. Importantly, the parameters included in the ABC stroke scoring system are widely used and highly operable.

### Early recurrence and late recurrence

Studies have shown that early recurrence of AF is a strong predictor of late recurrence [16]. The inflammatory response following cellular injury during catheter ablation induces abnormal conduction in atrial tissue and increases the susceptibility to arrhythmia. Additionally, the recovery of pulmonary vein conduction is a significant mechanism contributing to early recurrence, substantially elevating the likelihood of late recurrence [17]. This process may also reshape the atrial matrix by increasing sympathetic activation and reducing the vagus nerve activity thus altering the autonomic nervous system. Atrial remodeling, as a result, could generate new AF trigger foci, ultimately leading to ablation failure [18, 19]. The findings of this study substantiate the notion that early recurrence of AF stands as an independent risk factor for late recurrence.

### Clinical significance

Effective assessment of the risk of late recurrence of AF after RFCA is crucial for guiding treatment decisions for doctors and patients. The ABC stroke scoring system, known for its efficacy in evaluating stroke risk in patients with AF, can be employed to stratify the risk of late recurrence following AF ablation. The indicators within the ABC stroke scoring system are easy to obtain clinically and are easy to implement. It is worthwhile to further investigate whether controlling the factors incorporated in the ABC stroke scoring system, such as prevention of stroke and the reduction of preoperative NT-proBNP and troponin levels, can contribute to a reduction in late recurrence.

### Limitation

This study was a retrospective, single-center, limited number of patients study. Patients with organic heart disease such as cardiomyopathy and severe valvular disease, which are known to be associated with a higher likelihood of developing AF were excluded. The ABC stroke scoring system initially designed to evaluate stroke risk in patients with AF primarily emphasizes factors correlated with stroke. However, the value of other clinical indicators and biological markers in predicting late recurrence of AF after RFCA cannot be ignored.

## Conclusion

Patients with a higher ABC stroke score exhibit a higher rate of AF recurrence compared with those with a lower ABC stroke score. ABC stroke score ≥ 6.4 may serves as a predictor of increased late recurrence of PAF after RFCA. However, further studies are needed to elucidate the full value and potential of the ABC stroke scoring system in evaluating the prognosis of AF ablation.

## Data Availability

All data generated or analysed during this study are included in this article. Further enquiries can be directed to the corresponding author.

## Abbreviations

PAF: Paroxysmal Atrial Fibrillation
RFCA: Radiofrequency Catheter Ablation
NYHA: New York Heart Association
ROC: Receiver Operating Characteristic
AUC: Area Under Curve
BMI: Body mass index
ERAA: Early recurrence of atrial arrhythmia
HDL-C: High-density lipoprotein cholesterol
HTHD: Hypertensive heart disease
LAD: Left atrial diameter
LDL-C: Lowdensity lipoprotein cholesterol
LVEF: Left ventricular ejection fraction
TC: Total cholesterol
TG: Triglyceride
LVEDD: Left ventricular end-diastolic dimension
