# Correction: Influence of ABC stroke score on late recurrence of paroxysmal atrial fibrillation following radiofrequency catheter ablation

**DOI:** 10.1186/s13019-024-02944-z

**Published:** 2024-07-16

**Authors:** Ke-Zeng Gong, Zhe Xu, Ting-Pei Zhuang, Xue-Hai Chen, Jian-Hua Chen, Wei-Wei Wang, Wen-Hua Xu, Fei-Long Zhang

**Affiliations:** 1 Department of Cardiology, Fujian Clinical Medical Research Center for Heart and Macrovascular Diseases, Fujian Medical University Union Hospital, Fujian Heart Medical Center, Fujian Institute of Coronary Heart Disease, No.29 Xin-Quan Road, Gulou District, Fuzhou, Fujian 350001 China; 2 Department of Cardiology, The First Hospital of Quanzhou, Quanzhou, Fujian 362000 China; 3 Department of Cardiology, Changji Prefecture People’s Hospital in Xinjiang Uygur Autonomous Region, No.303 Yan-an Road, Changji City, Xinjiang 831100 China

**Correction: Journal of Cardiothoracic Surgery (2024) 19:344**

10.1186/s13019-024-02847-z

Following publication of the original article [1], in the Funding Section, the grant number relating to Medical Innovation Project of Fujian Health Commission was incorrectly given as 2021CX01010068 and should have been 2021CXB003.

The original article has been corrected.
